# Inherited Thrombophilia in the Era of Direct Oral Anticoagulants

**DOI:** 10.3390/ijms23031821

**Published:** 2022-02-05

**Authors:** Lina Khider, Nicolas Gendron, Laetitia Mauge

**Affiliations:** 1 Service de Médecine Vasculaire, Assistance Publique Hôpitaux de Paris-Centre (AP-HP.CUP), F-75015 Paris, France; lina.khider@aphp.fr; 2 Innovative Therapies in Haemostasis, Université de Paris, INSERM, F-75006 Paris, France; nicolas.gendron@aphp.fr; 3 Biosurgical Research Lab (Carpentier Foundation), AP-HP, F-75015 Paris, France; 4 Service d’Hématologie Biologique, Assistance Publique Hôpitaux de Paris-Centre (AP-HP.CUP), F-75015 Paris, France; 5 PARCC, Université de Paris, INSERM, F-75015 Paris, France

**Keywords:** inherited thrombophilia, direct oral anticoagulant, antithrombin, protein C, protein S, DOAC neutralization

## Abstract

Severe inherited thrombophilia includes rare deficiencies of natural anticoagulants (antithrombin and proteins C and S) and homozygous or combined factor V Leiden and FII G20210A variants. They are associated with a high thrombosis risk and can impact the duration of anticoagulation therapy for patients with a venous thromboembolism (VTE) event. Therefore, it is important to diagnose thrombophilia and to use adapted anticoagulant therapy. The widespread use of direct anticoagulants (DOACs) for VTE has raised new issues concerning inherited thrombophilia. Concerning inherited thrombophilia diagnosis, DOACs are directed toward either FIIa or FXa and can therefore interfere with coagulation assays. This paper reports DOAC interference in several thrombophilia tests, including the assessment of antithrombin, protein S, and protein C activities. Antithrombin activity and clot-based assays used for proteins C and S can be overestimated, with a risk of missing a deficiency. The use of a device to remove DOACs should be considered to minimize the risk of false-negative results. The place of DOACs in the treatment of VTE in thrombophilia patients is also discussed. Available data are encouraging, but given the variability in thrombosis risk within natural anticoagulant deficiencies, evidence in patients with well-characterized thrombophilia would be useful.

## 1. Introduction

Venous thromboembolic disease (VTD) is a multifactorial disease resulting from the interaction between environmental, clinical, and biological risk factors. Biological thrombophilia corresponds to the presence of an acquired or inherited biological risk factor predisposing the patient to venous thromboembolism (VTE) [1]. According to Jean Connors in a previous review on this topic [2]: “Ordering thrombophilia tests is easy; determining whom to test and how to use the results is not”. Thrombophilia assessment can be useful for the propositus and help in the assessment of the benefit/risk ratio in terms of the duration of anticoagulant treatment. In some cases, such as antithrombin (AT) deficiency, it can also be useful for symptomatic patients treated with heparin (increasing heparin doses to achieve therapeutic anticoagulation) and for asymptomatic relatives, especially women of childbearing age (prevention of thrombosis during pregnancy and in the case of estrogen pills). 

Classical inherited thrombophilia includes loss-of-function variants in the genes that encode the natural anticoagulant proteins, AT, protein C (PC), and protein S (PS), and gain-of-function mutations in the genes encoding factor V (factor V Leiden (FVL)) and prothrombin (FII) (*F2* c.*97G>A; previous nomenclature G20210A) [1]. Rarer congenital thrombophilias have been described in the literature, such as genetically determined increased homocysteine levels, coagulation factors (VIII and IX), and hypodysfibrinogenemia [2,3,4]. Patients with inherited hypodysfibrinogenemia can experience bleeding or thrombosis depending on the underlying sequence variation [4,5]. Moreover, growing data suggest an association between the non-O blood types and VTE [6,7]. The present review does not discuss acquired thrombophilia such as APS, focusing only on the most frequent inherited thrombophilia.

French guidelines recommend not performing a systematic constitutional thrombophilia test after the first episode of VTE [8], especially after the age of 50, whether or not the thrombosis is provoked. Thus, it is suggested to perform inherited thrombophilia testing in patients with a first episode of unprovoked proximal deep vein thrombosis (DVT) or pulmonary embolism (PE) before the age of 50 and with a first-degree family history of thrombosis, in patients with recurrent VTE (at least one episode of proximal DVT or PE and at least one unprovoked episode before age 50), or in patients with unprovoked VTE at atypical sites.

When inherited thrombophilia testing is indicated, it is suggested that you search for the following abnormalities: AT, PC, or PS deficiency and the presence of FVL (HGVS nomenclature: *F5* c.1601G>A variant) and the *F2* G20210A variant (HGVS: *F2* c.*97G>A) between the third and the sixth month after the VTE diagnosis [8]. In the case of confirmed natural inhibitor deficiency (AT, PC, and PS), the type of deficiency is characterized using complementary plasma tests, even with genotyping (see Section 2) [9]. Genotyping and family survey can help after a diagnosis of thrombogenic mutations not associated with frank plasmatic inhibitor deficiencies and in counseling for recessive forms, for example [10,11]. 

Treatment of VTE is based on the use of anticoagulants for a variable period according to different factors. Direct oral anticoagulants (DOACs), which have been available for over 10 years, include dabigatran, which inhibits thrombin, and apixaban, rivaroxaban, and edoxaban, which inhibit factor Xa (FXa). DOACs have demonstrated non-inferiority to vitamin K antagonists (VKAs) in treating acute symptomatic VTE with reduced risks of major bleeding [12,13]. They are taken orally, have a rapid onset of anticoagulation, and do not require routine monitoring. Several years ago, the use of DOACs increased when guidelines gave preference to them over VKAs [14]. 

In this context, we aim to explain which thrombophilia testing to perform in DOAC-treated patients while summarizing the available data concerning the efficacy of DOAC therapy in patients with inherited thrombophilia. 

## 2. Inherited Thrombophilia

The frequency and thromboembolic risk of an initial episode of thrombosis or a recurrence depend on the type of thrombophilia (severe or non-severe), as detailed in Table 1 [15,16,17]. Genetic and environmental factors also interact to bring about venous thrombosis. Combined defects have been described, such as the SERPINC1 variant and F5 Leiden cis-segregation, and are associated with a higher risk of thrombosis [18]. Common selected gene variants may also strongly synergize with less common mutations, leading to potential life-threatening conditions when combined with rare severest mutations [19].

### 2.1. FVL

FVL was first described in 1994 [20]. It results from the replacement of guanine by adenosine in position 1601 (old nomenclature 1691) of the F5 gene. Arginine (Arg) in position 534 (old nomenclature 506) of the coagulation cofactor V is thus replaced by a glutamine (Glu). Activated PC (APC) inhibits FVa by a cleavage at Arg 334 (previous nomenclature 306), 534 (506), and 707 (679). Therefore, FVL is resistant to APC inactivation, resulting in a gain of function of FVa [21,22]. The transmission of FVL is autosomal dominant. FVL heterozygosis is the most common prothrombotic gene variant in the Caucasian population, with a prevalence of approximately 5%, ranging from 3 to 7%, with a positive gradient from southern to northern Europe. FVL heterozygosis is present in the United States, with an average frequency of 5% in the general population. It is lower among Black Americans and Africans (1%) and Asians (0.5%) [1]. This prevalence rises to 20% in non-selected VTE patients. Homozygosity for FVL occurs approximately in 1 per 1000 individuals in the general population and in 1% of non-selected VTE patients. VTE risk increases approximately fivefold in heterozygous and tenfold in homozygous carriers. Moreover, individuals with homozygous FVL and/or homozygous *F2* c.*97G>A or double heterozygous carriers of FVL and *F2* c.*97G>A do not have a high risk of recurrent VTE [23].

### 2.2. F2 c.*97G>A Variant

The intronic substitution c.* 97G>A (previous nomenclature G20210A) of the F2 gene is located downstream of the sequence coding, in the 3′ untranslated region (3′ UTR). It is in a functional region that conditions the maturation of messenger RNAs. This variant increases the efficiency of cleavage and maturation, resulting in the accumulation of mature mRNA in the cytoplasm and an increase in protein synthesis [24]. This mechanism explains the significant association of the variant with approximately 30% higher levels of FII in heterozygous patients. By its impact on thrombin generation, this increase in concentration could explain the influence of this variant on the risk of thrombosis. Less prevalent than FVL, the F2 C.*97G>A variant is the second-most-common inherited thrombophilia, with a prevalence in Europe of approximately 2%, with an increasing southern–northern gradient. It is rarer in people from Africa and Asia. The transmission of the F2 C.*97G>A variant is autosomal dominant. The presence of this variant heterozygosity confers an approximately threefold increased risk for a first VTE event, though its presence has not consistently demonstrated any increased risk of recurrent VTE [1].

### 2.3. AT Deficiency 

AT is a serine protease inhibitor (serpin), a single-stranded glycoprotein of 464 amino acids (AA) synthesized by the liver. It is the main inhibitor of thrombin (activated factor II (FIIa)) and an important inhibitor of other coagulation serine proteases, particularly FXa. Thus, it reduces thrombin generation. In addition to the active site (reactive site (RS)) responsible for the coagulation factor inhibition, AT contains a heparin-binding site (HBS). The inhibitory function of AT is enhanced at least a thousandfold when exogenous heparin or heparin sulfate binds to this site. Currently, over 300 loss-of-function variants have been identified in the AT gene (*SERPINC1*) located on chromosome 1 (http://www.hgmd.cf.ac.uk (accessed on 2 October 2021)). AT deficiency is transmitted as an autosomal-dominant trait, and the penetrance of the disease is high since most affected family members present a thrombotic event by the age of 50. 

In 1965, AT deficiency was the first inherited thrombophilia identified [25]. The estimated prevalence of AT deficiency is extremely rare, from 0.02 to 0.2% in the general population. Patients with AT deficiency have a high-risk ratio for a first episode of VTE and carry a great risk of recurrent VTE.

AT deficiency is classified into two subtypes based on the plasma levels of the enzymatic activity and antigen (Ag). Type I (approximately 80% of cases) is a quantitative defect characterized by a reduced functional protein with a parallel reduction in the plasma AT Ag level. Type II is a qualitative defect characterized by a normal Ag level with impaired inhibitory activity reflecting the normal synthesis of a dysfunctional AT. In qualitative IIRS- or HBS-AT deficiency, the function of the RS and the HBS or the HBS only, respectively, is abnormal but the Ag level is normal (or slightly decreased but less than the activity). In IIPE-type deficiency (pleiotropic), the stability of the protein conformation is altered, which explains a slight decrease in AT activity and Ag or AT activity and Ag at the lower limit of reference values. According to the meta-analysis of observational studies by Di Minno et al. [16], the relative risk of a first episode of VTE associated with AT deficiency was 15 and the risk of recurrence was 4. These data are probably relatively inaccurate, not obtained from studies accounting for *SERPINC1* genotypes. Molecular bases have been established, and many variants of *SERPINC1* have been identified (http://www.hgmd.cf.ac.uk (accessed on 2 October 2021)). 

The clinical phenotype associated with AT deficiency is heterogeneous [26]. Type I AT deficiency is usually severe when there is a significant reduction in the AT plasma concentration. In types II, the heterogeneity is more important. In fact, on one hand, some *SERPINC1* variants result in clinical phenotypes as severe as type I deficiency by a negative dominant effect (i.e., variant IIRS, p.Arg425del, or conformational variants). On the other hand, other variants have a moderate functional effect and are associated with a less severe risk of thrombosis, such as the Cambridge II variant (p.Ala416Ser). HBS deficiency was previously associated with a lower risk of thrombosis. However, recent data have shown heterogeneity in the thrombosis risk within the HBS group and some variants appear as thrombotic as type I deficiencies, such as the Budapest III variant (p.Leu131Phe), even in heterozygous deficiency [26]. Homozygous AT deficiencies are extremely rare because they are most often lethal before birth and are only encountered for type IIHBS deficiencies (e.g., Budapest III and p.Arg79Cys) or other variants relatively less harmful (e.g., Cambridge II and Dublin p.Val30Glu). *SERPINC1* genotyping cannot explain all the constitutional abnormalities of AT; rare inherited AT deficiencies are explained by glycosylation abnormalities in the context of the congenital disorder of glycosylation (CDG) syndrome [27].

### 2.4. PC Deficiency

PC is a vitamin-K-dependent plasma glycoprotein synthesized by the liver as a zymogen of serine protease. PC is activated by thrombin. This process is enhanced by the complex formed by thrombin and thrombomodulin and the binding of PC on its endothelial PC receptor. With PS, activated PC (APC) inhibits thrombin generation by proteolytic inactivation of coagulation cofactors FVa and FVIIIa. The PC pathway plays a significant role in regulating the thrombotic process, especially in microcirculation, where the contact between the proteins and the endothelium is important.

The spectrum of clinical manifestations caused by PC deficiency is relatively broad. The phenotypic heterogeneity of PC deficiency may reflect heterogeneity at the molecular level. The molecular basis for PC deficiency has been established, and over 200 loss-of-function mutations have been identified in the PC gene (PROC) located on chromosome 2 (http://www.hgmd.cf.ac.uk (accessed on 2 October 2021)).

Usually, PC deficiency is thought to be transmitted as an autosomal dominant trait with a high degree of penetrance, but in families with individuals with complete deficiency, the mode of inheritance has been classified as autosomal recessive. The prevalence of PC deficiency is 0.2% to 0.4% in the general population and 3.0% in unselected VTE patients.

PC deficiency is classified based on the plasma levels of the enzymatic activity and the Ag level, divided into two subtypes. Type I, the most frequent, is characterized by a parallel reduction in the plasma level of Ag and activity, reflecting a reduced synthesis of a functional PC. Type II is rare, characterized by normal Ag but reduced activity, reflecting the normal synthesis of a dysfunctional PC. PC dysfunction can concern the enzymatic function of PC (type IIa (IIAM), or amidolytic deficiency) or the procoagulant function of PC not associated with the enzymatic activity (type IIb (IIAC), or anticoagulant deficiency; Table 2). Heterozygous PC deficiency is the most frequent. Individuals with complete PC deficiency (homozygous) or those with extremely low levels of plasma PC have a more severe clinical picture, sometimes revealed by purpura fulminans in newborns, a potentially fatal condition characterized by microvascular thrombosis and skin necrosis [28,29]. PC deficiency increases the risk of VTE [30,31]. In a meta-analysis of observational studies by Di Minno et al. [16], the relative risk of a first episode of VTE associated with PC deficiency was 7 and the risk of recurrence was 3 [16]. These data are probably relatively inaccurate, not obtained from studies accounting for *PROC* genotypes [11].

### 2.5. PS Deficiency 

PS is a vitamin-K-dependent glycoprotein with a circulating form of 70 kDa comprising 635 AAs. The synthesis of PS does not exclusively occur in the liver; it is produced by endothelial cells, megakaryocytes, Leydig cells, and the brain. The gene encoding PS (*PROS1*) is located on chromosome 3. The N-terminal part of the mature protein contains a GLA domain that binds calcium ions, and whose presence conditions the affinity of PS for membrane phospholipids, followed by a sensitive loop to thrombin. The C-terminal part includes a domain binding to the C4b binding protein (C4bBP). In plasma, PS circulates mainly in two forms, one free form (40%) and one bound (60%) to C4bBP. Free PS is a non-enzymatic cofactor of APC for the proteolysis of FVa and VIIIa. Moreover, PS has anticoagulant activities independent of APC, i.e., as a cofactor of the tissue factor pathway inhibitor in the inhibition of FXa.

Inherited PS deficiency is transmitted as an autosomal dominant trait. Its prevalence in the general population could be between 0.03% and 0.13%. According to a meta-analysis of observational studies by Di Minno et al. [16], the relative risk of a first episode of VTE associated with PS deficiency was 5 and the relative risk of recurrence was 2.5 (but not significant). These data are probably relatively inaccurate, not obtained from studies accounting for *PROS1* genotypes [10]. Homozygous PS deficiencies with the absence of circulating protein have been reported. The symptomatology is identical to that of the homozygous PC deficiencies. Several studies have shown that PS-inherited deficiencies associated with an increased risk of thrombosis are those for which plasma-free PS is greatly reduced, with a risk threshold (30-40%) lower than the lower limit of the reference values [10]. PS deficiency can be classified as quantitative (types I and III) and qualitative (rarer) types. Type I is a quantitative deficiency characterized by decreased plasma levels of functional and Ag total or free PS. Type III is also a quantitative deficiency with reduced functional activity and free PS but normal total PS levels [1]. Type I and III deficiencies can be phenotypic variants of the same genetic change, while type II qualitative deficiencies are associated with a normal level of total and free PS and a decreased APC cofactor activity. 

The molecular basis for PS deficiency has been established, and almost 200 mutations of *PROS1* have been identified (http://www.hgmd.cf.ac.uk (accessed on 2 October 2021)). *PROS1* genotyping does not explain all inherited abnormalities of PS. Thus, PS deficiency linked to constitutional glycosylation abnormalities may be associated with thrombotic events as described in CDG syndrome. Data concerning genotype–phenotype relationships are available [32]. All variants do not appear to be equivalent in terms of the risk of VTE. Thus, type II variants could be less thrombotic than quantitative type variants [33]. The presence of a deleterious quantitative mutation and the level of circulating PS have a synergic influence on the risk of VTE [10]. The Heerlen variant (p.Ser501Pro, HGVS nomenclature) alters a glycosylation site, reducing its circulating half-life with a modest decrease in the plasma PS level. Data on the VTE risk associated with the Heerlen mutation are contradictory [10,34,35].

## 3. Laboratory Testing for Inherited Thrombophilia upon DOACs

### 3.1. Biological Diagnostics of Inherited Thrombophilia

The detection of FVL was initially based on plasmatic testing searching for an APC resistance. Because of a lack of sensitivity and the absence of information on the genetic status, molecular diagnosis is now indicated as a first-line test [36]. Detection of the G20210A variant of *F2* (c.*97G>A, HGVS) is also based on molecular biology. These analyses are not impacted by the presence of treatment and can be realized in patients treated with DOACs. Because APC resistance testing is no longer recommended to search for FVL, the influence of DOACs on this analysis is not detailed in this review. 

Screening for the inherited deficiency of natural anticoagulants (AT, PC, and PS) is based on functional tests assessing anticoagulant activity to detect all types of deficiencies, both quantitative and qualitative [37]. Screening for AT deficiency is based on chromogenic tests assessing heparin cofactor activity to inhibit FXa or FIIa. If a decrease is observed and not explained by an acquired etiology (e.g., consumption or liver dysfunction), the quantification of an antithrombin antigen will guide toward a quantitative or qualitative deficiency (Table 2). For PC and PS, clot-based anticoagulant activity testing can detect all types of deficiencies. Many factors can interfere with these tests (biological parameters, including the level of FVIII and treatments); hence they are not always used as first-line tests. However, they are indispensable in detecting type IIb PC and type II PS deficiencies. The diagnosis of PC deficiencies also includes a chromogenic test that assesses PC enzymatic function and antigenic assays. PS deficiency diagnosis is completed by a PS-free Ag measurement. Genotyping is helpful for the identification of qualitative AT deficiency and its associated thrombotic risk. It can be useful to confirm PC or PS deficiencies. The place and techniques for genotyping are not discussed here as DOACs do not interfere with molecular biology.

Because of the acquired transient modification of AT, PC, and PS that can be encountered during the acute phase of thrombosis, it is recommended to realize thrombophilia testing between the third and sixth months after a VTE event [8]. However, the results can impact the treatment duration. Therefore, thrombophilia testing can occur while the patient is on anticoagulant treatment. For each inhibitor, we detail the reported interferences of DOACs in the tests used to detect inherited deficiencies and the results of DOAC neutralization for thrombophilia testing. 

### 3.2. Interference of DOACs in Inherited Thrombophilia Testing

#### 3.2.1. Antithrombin

AT inhibitory activity is usually assessed by measuring residual FXa or FIIa activity with the addition of an excess amount of the appropriate enzyme to the test plasma (chromogenic assays). Accordingly, FXa or thrombin is inhibited in these assays, not only by AT but also by DOACs, thus explaining the overestimation of AT inhibitory activity. 

Methods based on FXa inhibition are affected by rivaroxaban [38,39,40,41,42,43,44,45], apixaban [40,41,43,44,45,46,47,48,49], and edoxaban [41,49,50], whereas those based on FIIa inhibition are affected by dabigatran [39,44,51,52,53,54,55,56,57] (Table 3). The overestimation is dose dependent [43,44,49,52] and varies depending on the DOACs and on the reagents [41,52]. In the studies realized with DOAC-spiked plasma, some assays were not impacted when the DOAC concentration was low. Examples of an increase in AT activity reported are 12% per 200 ng/mL of dabigatran with Stachrom ATIII (IIa-based assay) [53], 13% per 100 ng/mL of apixaban with HemosIL (Xa-based assay) [47], 3% per 100 ng/mL of edoxaban with Innovance AT (Xa-based assay) [49], and 9% per 100 ng/mL of rivaroxaban with Coamatic AT LR (Xa-based assay) [38]. No significant influence was observed with the Innovance AT reagent when the rivaroxaban plasma concentration was <100 ng/mL [41]. Similarly, dabigatran concentrations < 100 ng/mL did not seem to influence FIIa-based AT activity when measured with the Stachrom ATIII reagent [52,54,55]. Notably, the statistical estimation of an absence of DOACs’ impact on an assay was heterogeneous. 

Most studies were conducted on pooled normal plasma spiked with different concentrations of DOACs. Others used plasma from DOAC-treated patients and compared the results with those obtained with a non-impacted assay, for example, results from FIIa-based assays for patients treated with anti-Xa DOAC [43], or compared the results with baseline levels of AT before introducing the DOAC treatment [42]. The results of one ex vivo study differed from the observations made with normal pooled spiked plasma. No significant interference of dabigatran in the measure of AT activity with anti-IIa based assays was observed by Favresse et al. [57]. This finding can be explained by the low median concentration of dabigatran in these patients (73.5 ng/mL). 

#### 3.2.2. Protein C

Clot-based assays for PC rely on the inhibition of clot formation following PC activation. Whatever the way to activate the coagulation, activated partial thromboplastin time (aPTT) or Russel’s viper venom (RVV) assays, FXa and FIIa are involved in clot formation. The prolongation of the clotting time is proportional to the concentration of PC in the plasma sample. Any DOAC can affect those tests, leading to an overestimation of the PC anticoagulant activity (Table 3) [41,46,49,51,58], which has the potential to mask a state of deficiency.

A dose-dependent overestimation of the PC anticoagulant activity has been described with apixaban and rivaroxaban [49,58]. For example, an increase in the PC activity of 4% per 100 ng/mL of apixaban has been reported with Staclot PC (clot-based assay) [49]. The sensitivity of PC clot-based assays to DOACs varied depending on the drugs and the reagents used. Overestimation was less elevated with apixaban than with rivaroxaban [58]. A PC Coag assay was less sensitive to apixaban than Staclot Protein C [49]. Interestingly, no significant impact of DOACs was reported for low concentrations of dabigatran (<100 ng/mL) [51] and even high concentrations of apixaban (>750 ng/mL), edoxaban (>276 ng/mL), and rivaroxaban (>222 ng/mL) with the PC reagent from Siemens [41].

Most studies were conducted on pooled normal plasma spiked with different concentrations of DOACs. Contrary to AT, all types of DOACs impacted PC clot-based assays. Currently, no equivalent alternative strategy can be proposed. Therefore, demonstrating the influence of DOACs using the plasma from DOAC-treated patients can be more difficult. Notably, fewer data are available on the influence of DOACs on clot-based PC assays than on AT activity or clot-based PS assays. 

As expected, the measurement of antigenic PC or functional PC by a chromogenic method was unaffected by DOACs (Table 3) [40,41,42,45,47,49,50,51,52,54,55,56,57,59]. Many studies have tested the chromogenic assays on spiked-pooled normal plasma. 

#### 3.2.3. Protein S

Functional (clot-based) PS assays are based on either prothrombin time (PT), aPTT, or RVV. They rely on the inhibition of clot formation by PS in the presence of APC, FXa, and FIIa, involved in clot formation, whatever the assay. The prolongation of the clotting time is proportional to the concentration of PS in the plasma sample. Samples containing DOAC will prolong the clotting time, leading to overestimated PS activity and the potential miss of a deficiency (Table 3) [40,41,46,47,49,50,51,52,54,55,58,59,60,61,62]. 

A dose-dependent overestimation of PS anticoagulant activity was described with dabigatran, apixaban, and rivaroxaban [40,52,58]. For example, an increase in PS activity of 15% per 100 ng/mL of rivaroxaban was reported with Staclot PS (clot-based assay) [54]. The sensitivity of PS clot-based assays to DOACs varied depending on the drugs and the reagents used. The impact of DOACs was reported for low concentrations of dabigatran [51,55] and rivaroxaban [41]. Overestimation was less elevated with apixaban than with rivaroxaban [58]. The impact of DOACs seemed more important on PS clot-based assays based on PT or RVV activation than on aPTT-based assays [63]. 

Most studies were conducted on pooled normal plasma spiked with different concentrations of DOACs. Contrary to AT, all types of DOACs impact PS clot-based assays. No equivalent alternative strategy can be proposed. Therefore, assessing the impact of DOACS on plasma from treated patients is more difficult. Maryamchik et al. compared the mean PS functional activity with that of the mean free PS Ag in 32 patients treated with rivaroxaban (145 ng/mL; range 23 to 349 ng/mL) and in 40 patients treated with apixaban (139.8 ng/mL; range 27.5 to 652.0) [60,62]. Rivaroxaban falsely elevated PS activity and caused missed diagnoses of PS deficiency, whereas with patients on apixaban, functional PS activity was not significantly different from that of free PS Ag. Furthermore, low free PS Ag occurred in 3 of 40 (8%) patients in this study, and in all three cases, PS activity was also low during apixaban treatment. These results are in line with Gosselin et al. reported apixaban interference only for concentrations above 471 ng/mL [41]. Different results were obtained by Hillarp et al., who described an increase of 13% in PS activity for low concentrations of apixaban (<100 ng/mL) [49]. Mani et al. also used plasma from rivaroxaban-treated patients. They compared the results to baseline levels of PS measured before introducing the DOAC treatment [42]. 

Immunologic PS assays are usually determined using ELISA-based assays or latex-particle-based agglutination assays. As expected, the measurement of antigenic free PS is not affected by DOACs [40,45,45,49,50,51,52,54,56,57,57,60,61] (Table 3).

**Table 3:** Interference of DOACs in inherited thrombophilia testing.

Methods	Dabigatran	Apixaban	Edoxaban	Rivaroxaban
**Antithrombin activity**				
FXa-based assays	**√ no effect**	**X increase**	**X increase**	**X increase**
	➢ In vitro studies: 25–1000 ng/mL 5 assays tested [39,51,52,53]	➢ In vitro studies: 8–1000 ng/mL 5 assays tested [40,41,47,48,49]	➢ In vitro studies: 10–600 ng/mL 3 assays tested [41,49,50]	➢ In vitro studies: 7–1000 ng/mL 4 assays tested [38,39,40,41]
	➢ Ex vivo studies: 17 ng/mL (IQR, 48–144) (*n* = 27) 1 assay tested [44]	➢ Ex vivo studies: 94 ng/mL (IQR, 64–145) (*n* = 54)/NP (*n* = 72) 1 assay tested [43,44]		➢ Ex vivo studies: 104 ng/mL (IQR, 45–334) (*n* = 49)/NP (*n* = 86)/prophyactic (*n* = 47) 2 assays tested [42,43,44]
FIIa-based assays	X increase/no effect*	**√ no effect**	**√ no effect**	**√ no effect**
	➢ In vitro studies: 4.7–1000 ng/mL 4 assays tested [39,51,52,53,54,55]	➢ In vitro studies: 8–1000 ng/mL 2 assays tested [40,47,48,49]	➢ In vitro studies: 10–500 ng/mL 2 assays tested [49,50]	➢ In vitro studies: 7–1000 ng/mL 3 assays tested [38,39,40,59]
	➢ Ex vivo studies: 71 ng/mL (IQR, 48–144) (*n* = 27)/2–406 ng/mL (*n* = 30) 2 assays tested [44,57]	➢ Ex vivo studies: 94 ng/mL (IQR, 64–145) (*n* = 54)/NP (*n* = 72)/10–316 ng/mL (*n* = 26) 3 assays tested [43,44,57]	➢ Ex vivo studies: 21–354 ng/mL (*n* = 10) 1 assay tested [57]	➢ Ex vivo studies: 104 ng/mL (IQR, 45–334) (*n* = 49)/NP (*n* = 86)/prophylactic (*n* = 47)/7–457 ng/mL (*n* = 27) 3 assays tested [42,43,44,57]
**Protein C activity**				
Clot-based assays	**X increase**	**X increase**	**X increase**	**X increase**
	➢ In vitro studies: 25–500 ng/mL 1 assay tested [51]	➢ In vitro studies: 10–1000 ng/mL 2 assays tested [41,49]	➢ In vitro studies: 10–~600 ng/mL 1 assay tested [41]	➢ In vitro studies: 10–~600 ng/mL 1 assay tested [41]
Chromogenic assays	**√ no effect**	**√ no effect**	**√ no effect**	**√ no effect**
	➢ In vitro studies: 10–800 ng/mL ≥3 assays tested [51,52,55]	➢ In vitro studies: 8–1000 ng/mL ≥3 assays tested [40,41,47,49]	➢ In vitro studies: 10–~600 ng/mL 2 assays tested [41,50]	➢ In vitro studies: 7–638 ng/mL ≥3 assays tested [40,41,54,59]
				➢ Ex vivo studies: prophylactic 1 assay tested [61]
**Protein S activity**				
Clot-based assays	**X increase**	**X increase/no effect**	**X increase**	**X increase**
	➢ In vitro studies: 10–800 ng/mL ≥2 assays tested [51,52,55]	➢ In vitro studies: 8–750 ng/mL ≥2 assays tested [40,41,47,49]	➢ In vitro studies: 10–600 ng/mL 1 assay tested [41,50]	➢ In vitro studies: 7–638 ng/mL ≥1 assay tested [40,41,54,59,63]
		➢ Ex vivo studies: 140 ng/mL (range, 28–652) 1 assay tested [60]		➢ Ex vivo studies: prophylactic (*n* = 40)/145 ng/mL (range, 23–349) 1 assay tested [61,62]
**Protein S Ag**				
Protein S free Ag	**√ no effect**	**√ no effect**	**√ no effect**	**√ no effect**
	➢ In vitro studies: 25–800 ng/mL ≥2 assays tested [49,51,52]	➢ In vitro studies: 8–501 ng/mL 2 assays tested [40,47]	➢ In vitro studies: 100–500 ng/mL 1 assay tested [50]	➢ In vitro studies: 7–638 ng/mL 2 assays tested [40,54]
		➢ Ex vivo studies: 140 ng/mL (range, 26–652) 1 assay tested [60]		➢ Ex vivo studies: prophylactic (*n* = 40) 1 assay tested [61]

NP: not precised. Testings which can be impacted by the presence of DOAC leading to an overestimation of natural inhibitors are marked by “**X**”. Others by “**√**”.

Thrombophilia testing may be significantly affected by even small amounts of DOACs. Their effects are drug, concentration, test methodologic, and reagent dependent. AT activity can be studied with either FXa- or FIIa-based assays depending on the target of the DOACs. For PC and PS, apixaban apparently interferes less than rivaroxaban. PC clot-based assays would be less sensitive to DOACs, with a possible testing at low concentrations that must be confirmed for each reagent.

### 3.3. DOAC Neutralization

Several strategies were proposed to minimize the impact of residual DOACs on coagulation assays. Missing one (for once-daily fixed-dose regimens) or two (for twice-daily fixed-dose regimens) doses of DOAC intake in patients with low thromboembolic risk has been proposed. However, except for apixaban, even DOAC trough levels may significantly impact thrombophilia testing. Therefore, to obtain accurate results, whenever possible, hemostasis testing should be performed four days after the DOAC treatment is discontinued; however, it is not a recommended clinical point of view. Switching from DOACs to low-molecular-weight heparin for a transient period may be an alternative when testing must be completed. DOAC-insensitive assays have been developed for lupus anticoagulant testing, but the same is not available for inherited thrombophilia testing. Specific reversing agents, such as idarucizumab against dabigatran and andaxanet alfa against rivaroxaban and apixaban have been developed, but they are expensive and have not yet become routine reagents used for diagnostic laboratories [64,65]. Ciraparantag is in clinical trials as a universal reversal agent for both FIIa and anti-Xa inhibitors. However, none of these approaches is optimal, and a simple way to overcome the problem is to remove DOAC from the plasma sample without influencing its coagulant property.

The means to avoid this interference is now available with the arrival of adsorbent agents able to remove DOAC from a plasma sample. Two devices are based on the use of activated charcoals. DOAC-Stop^®^ (Haematex Research) was the first device to remove DOACs from a plasma sample. A similar product, DOAC remove^®^ (5-Diagnostic AG), is also an absorbent tablet to put into plasma. According to the manufacturer, one tablet is added per 1 mL of plasma and mixed for 5 or 10 min. After centrifugation, the plasma is decanted to process the coagulation tests. Activated charcoals (AC) are porous elemental carbons with a large internal surface area. Most are commercially prepared products used in industry or in pharmaceuticals. Medical grade activated charcoal usually coated with porous hydrophilic polymers is more biocompatible with blood but may have a lower capacity for binding drug molecules to be adsorbed. Given the variable quality of ACs available from chemical suppliers, it seems unlikely they can be used interchangeably. More recently, filters coated with absorbent products, such as DOAC-Filter^®^ (Diagnostica Stago), have been developed [66]. DOAC-Filter^®^ is a ready-to-use single cartridge containing a solid phase. The solid-phase extraction principle is based on a noncovalent binding mechanism. The hydrophilic–hydrophobic balance of the solid phase has been determined to specifically trap DOACs. This device is easy to use and avoids the passing of residues potentially interfering with the measurement. There is a slight loss of volume after centrifugation. Basic requirements include an adequate binding capacity to handle the highest DOAC concentrations likely to be encountered clinically and not to impact testing in plasma without DOACs. Table 4 summarizes the performance of these devices concerning inherited thrombophilia testing.

All three processes can neutralize DOACs in plasma samples from treated patients. In addition, incomplete reversal was reported for 2 patients among 21 with DOAC Remove^®^ and 1 among 18 with DOAC-Filter^®^ [58,66]. The incomplete reversal did not always concern patients with the highest plasma DOAC concentrations.

Several groups have studied the impact of plasma treatment with these reversal agents on AT, PC, and PS levels. This impact was assessed using either plasma samples from non-treated healthy individuals [45,57,58,66,67] or methods of measurement not influenced by DOACs, such as an anti-FIIa-based assay to measure AT activity in patients treated with apixaban [44] or PC chromogenic assays [45,56,67]. No clinically relevant impact of DOAC neutralization was observed on AT, PC, or PS levels (Table 4). A significant decrease in PC amidolytic activity was reported by Monteyne et al. but only when using two tablets of DOAC-Stop^®^, which was not associated with a clinically relevant effect [67]. They also mentioned a decrease in PS levels (free PS Ag) in healthy individuals with both DOAC-removing agents when using the Wilcoxon rank test. The clinical impact was not precised, but the decrease seemed slight [67].

Studies on DOAC reversal were based on plasma from DOAC-treated patients. They reported a decrease in the inhibitor level after plasma treatment with the AC devices and confirmed the interference observed with normal plasma spiked with DOAC: interference in the clot-based measure of PC [58] and PS [58,63] and interference of anti-Xa DOACs in the measure of AT activity with FXa-based assays [44]. Smock et al. also reported a difference in the interference of dabigatran in PS clot-based assays depending on the method (an RVV- versus aPTT-based method) [63].

DOAC interference results in a possible overestimation of the results with the risk of masking a deficiency. When the measured level of AT, PC, or PS is low, a deficiency cannot be missed, since there is a risk of having a more profound defect than what is observable. However, when the level measured is normal, it is more difficult to know if a deficiency is masked. Thus, despite a decrease in the anticoagulant level observed when DOACs are neutralized, it can be difficult to establish the complete normalization of the result after DOAC reversal. Indeed, even at a concentration lower than the limit of quantification, DOAC can still interfere with some assays and mask a deficiency. For AT testing, two types of assays are available that can help to conclude on the correction brought by DOAC reversal. Zabcyck et al. investigated 130 patients who were on DOACs due to thromboembolism: 49 on rivaroxaban, 104 ng/mL (IQR, 45–334 ng/mL); 54 on apixaban, 93.5 ng/mL (IQR, 64–145 ng/mL); and 27 on dabigatran, 71 ng/mL (IQR, 48–144 ng/mL) [44]. Ten patients presented with AT deficiency detected with an anti-IIa based assay and genetically confirmed. All were treated with anti-FXa DOACs. With the anti-FXa based assay, AT activity was decreased only in the case of 3 patients. The DOAC-Stop^®^ treatment resulted in an increased number of patients with AT activity lower than reference values with the FXa-based assay, from 3 to 10 subjects. Hence, DOAC-Stop^®^ corrected the identification of the 7 false-negative patients with AT deficiency (4 treated with apixaban and 3 with rivaroxaban). Favre et al. investigated 47 patients treated with DOACs for whom thrombophilia testing was prescribed [58]. Inherited thrombophilia was screened in 12 patients treated with apixaban (plasma concentration: 165 ng/mL; range 80–634) and 9 with rivaroxaban (165 ng/mL; range 80–634). They compared PC and PS clot-based activities with patient Ag levels and described a dose-dependent overestimation induced by DOACs. Following the ex vivo treatment of plasma by DOAC Remove^®^, functional PC and PS overestimation returned to the same level as Ag measures. Moreover, a decrease in PS activity from 92% to 53% in one sample revealed a true PS deficiency, confirmed by the molecular analysis of the *PROS1* gene.

The adsorbent procedure appeared to be an effective and simple way to overcome the interference of DOAC in coagulation tests and should facilitate the interpretation of thrombophilia screening tests in patients taking DOACs. Compared to specific reversal agents, such as idarucizumab or andexanet alfa, the advantages of these procedures are the removal of all types of DOACs and that they are be simple, cheap, and easily accessible. A cutoff assuring an absence of interference needs to be established with DOAC-treated plasma. This cutoff must be specific of the reagent used and patient treatment. When clot-based assays are not used to assess the presence of PC or PS deficiencies, the possibility of missing a qualitative deficiency must be reported to the clinicians. 

## 4. Place of DOAC for the Treatment of VTD in Patients with Inherited Thrombophilia

DOACs are now widely used to prevent and treat VTD, and their indications are expanding over time. Numerous randomized clinical trials involving large numbers of patients have demonstrated the efficacy and safety of DOACs as alternatives to VKAs for the initial treatment and secondary prophylaxis of VTE. As explained before, severe thrombophilia groups rare biological disorders, which predisposes one to a higher incidence of VTE and a thrombosis risk depending on the type of deficiency (i.e., quantitative, qualitative, and genotype) and its severity. Given the significant advantages of DOACs over VKAs, there is a need for evidence of DOACs’ safety and efficacy in patients with severe thrombophilia. Below are reported observations of case reports, retrospective or prospective studies, on small cohorts, with sub-analyses from large studies, focusing on patients with severe thrombophilia, offering reassuring conclusions concerning DOAC efficacy in this population. 

Several case reports have described patients who presented with VTE in the context of severe thrombophilia, such as PS deficiency, and were successfully treated with edoxaban [68,69] or rivaroxaban [70,71,72,73,74]; PC deficiency successfully treated with rivaroxaban [75,76] or edoxaban [77,78]; AT deficiency successfully treated with apixaban [79] or rivaroxaban [80]; homozygous FVL mutation successfully treated with rivaroxaban [81]; or double heterozygous FVL and *F2* c.*97G>A mutations successfully treated with apixaban [82]. One case of a patient with severe hypofibrinogenemia successfully treated with rivaroxaban shows how challenging therapeutic management can become during the perioperative period [83].

Furthermore, several cohorts have published the efficacy and safety of DOACs in patients with thrombophilia. A recent prospective cohort of 56 consecutive patients with documented symptomatic venous thrombosis and/or PE [84] included 18 patients with AT deficiency, 12 with PC deficiency, 12 with PS deficiency, 4 patients with homozygous F2 C.*97G>A mutation, 3 patients with homozygous FVL, and 7 patients with combined heterozygous FVL and F2 C.*97G>A mutations. During a median follow-up of 44.5 (interquartile 30.0–52.5) months, rivaroxaban was used in 30 (53.6%) patients, apixaban in 14 (25.0%), and dabigatran in 12 (21.4%). Recurrent non-fatal VTE occurred in 5 (8.9%) patients, 4 of whom were treated with rivaroxaban and 1 with apixaban. Of these, 3 patients had combined defects consistently associating a heterozygous F2 C.*97G>A. Major bleeding occurred in 2 (3.5%) patients both with AT deficiency and clinically relevant non-major bleeding was observed in 4 (7.0%) patients mostly on rivaroxaban. This study showed a slight excess risk of bleeding in patients treated with rivaroxaban compared to those treated with dabigatran or apixaban (HR 2.76; 95% CI 1.26–3.92; *p* = 0.039).

In 2021, Hekman et al. [85] published a monocentric retrospective cohort study of patients with a diagnosis of acute lower extremity DVT with systematic follow-up by venous duplex ultrasound of the lower limbs. Among 496 patients with acute lower extremity DVT, 34% (*n* = 149) received DOACs, 28.3% (*n* = 140) had prior DVT, and 14.1% had prior PE. Only 7.1% of the population had a documented history of inherited or acquired thrombophilia. Treatment failure was defined as any new DVT or progression of an existing DVT within 3 months of diagnosis. A history of thrombophilia was not correlated with anticoagulant treatment failure. 

The START registry [86] is an observational, multicenter, dynamic cohort study that includes adult patients starting anticoagulation therapy. This registry includes 4,866 patients with acute VTE treated with therapeutic anticoagulant to treat and prevent VTE recurrence. Of these, 446 patients had thrombophilia, with 22 (4.9%) patients with AT deficiency, 28 (6.3%) with PC deficiency, 41 (9.2%) with PS deficiency, 101 (22.6%) with heterozygous or homozygous F2 C.*97G>A mutation, 205 (46.0%) patients with heterozygous or homozygous FVL mutation, and 49 (11.0%) patients with combined thrombophilia. Among the thrombophilia patients, 172 (38.5%) were under conventional anticoagulant therapy and 274 (61.5%) were under DOACs. This subgroup had significantly fewer clinically relevant non-major bleeding events in patients treated with DOACs vs. the conventional anticoagulant therapy group (3 (1.1%) vs. 9 (5.2%); *p* = 0.01), no difference in major bleeding (3 (1.1%) vs. 3 (1.8%)), and no difference in thrombotic events (3 (1.1%) vs. 1 (0.6%)). 

An Italian prospective cohort study [87] evaluated the efficacy and safety of DOACs in 45 patients with severe thrombophilia: 18 (40%) had PS deficiency, 5 (11.2%) had PC deficiency, 5 (11.2%) had AT deficiency, 12 (26.6%) had homozygous FVL mutations, and 4 (8.8%) had combined heterozygous FVL and F2 C.*97G>A mutations. Of these patients, 33 were switched to DOACs after an initial treatment with VKAs because of a fluctuating international normalized ratio (INR), difficulty in carrying out a regular monitoring, or patient request and 12 received DOACs as first-line treatment for VTE. The median VKA treatment follow-up was 60 months (range 6–180), and the median follow-up after DOAC initiation was 29 months. Rivaroxaban (44.4%) and apixaban (40%) were the most frequently used agents. No patients experienced bleeding or thrombotic complications during DOAC therapy, whereas during VKA treatment, the cohort observed three minor hemorrhagic complications and two thrombotic events. 

Similarly, a Polish cohort study [88] evaluated DOACs in 33 patients with severe thrombophilia. In this cohort, 5 patients had AT deficiency, 3 had PC deficiency, 4 had PS deficiency, 1 had homozygous FVL mutations, 3 had homozygous F2 C.*97G>A mutations, and 17 had combined defects. Rivaroxaban was used in 23 (70%) patients, while dabigatran and apixaban were used in 4 (12%) patients each. Two patients switched between DOACs. During a median follow-up of 21 months (range 8–34), three episodes of recurrent VTE (9%) were observed. All 3 patients continued to use DOACs: apixaban, dabigatran, and rivaroxaban. Lower efficacy was observed in PS deficiency, mainly related to non-adherence.

Finally, Campello et al. [89] presented a prospective cohort study showing that DOACs are effective and safe for treating VTE in patients with inherited thrombophilia although they carry an increased risk of clinically relevant non-major bleeding compared to heparin/VKA. Of 597 patients, 197 (33%) had severe thrombophilia, including 29 (14.7%) with PS deficiency, 15 (7.6%) with PC deficiency, 13 (6.6%) with AT deficiency, 9 (1.5%) with homozygous FVL mutations, 1 (0.2%) with homozygous *F2* c.*97G>A mutations, and 4 (8.8%) with combined heterozygous FVL and *F2* c.*97G>A mutations. In the DOAC group, 168 (61.1%) patients were treated with rivaroxaban, 55 (20%) with apixaban, 36 (13.1%) with edoxaban, and 16 (5.8%) with dabigatran. The cumulative incidence of recurrence during the anticoagulation period was 1.09% (95% CI 0.22–3.31) in the DOAC group versus 1.83% (95% CI 0.74–4.3) in the VKA/heparin group (adjusted HR 0.67 (95% CI 0.16–2.77)). The cumulative incidence of bleeding during the anticoagulation period was 10.2% (95% CI 7.09–14.36) in the DOAC group versus 4.97% (95% CI 3.02–7.97) in the VKA/heparin group (adjusted HR 2.24 (95% CI 1.10–4.58)). In contrast, no major bleeds were reported in the DOAC group. This study was a cohort of more than 500 patients with thrombophilia treated with DOACs that found no significant difference in the risk of recurrence but a slight increase in the number of bleeding events in the DOAC group. 

Using MarketScan claims data from January 2012 to September 2015, Coleman et al. identified adults diagnosed with a first episode of VTE during a hospitalization/emergency department visit and outpatients for whom a treatment with rivaroxaban or warfarin was initiated within 30 days of the index VTE [90]. They identified patients with baseline thrombophilia, including FVL and F2 C.*97G>A mutations and PC, PS, and AT deficiencies, based on a coding algorithm highly specific for identifying (ruling in) laboratory-diagnosed primary hypercoagulable state but that may not have always corresponded with clinical diagnosis [91]. Using the propensity score, they matched 403 patients receiving rivaroxaban and 403 patients on VKAs. There was no statistical difference in recurrent VTE and major bleeding between both groups, with HRs of 0.70 (95% CI 0.33–1.49) and 0.55 (95% CI 0.16–1.86), respectively.

Moreover, Elsebaie et al. [92] published a meta-analysis of eight randomized clinical trials of patients treated with DOACs for VTE: EINSTEIN-DVT, EINSTEIN-PE, RE-COVER, RECOVER II, RE-MEDY, RAPS, and TRAPS. In each study, patient data on thrombophilia were extracted as described in the subgroup analyses. The risk of recurrence was assessed for 1994 patients with known thrombophilia, such as acquired APS or inherited. The meta-analysis found no significant difference in VTE recurrence rates between patients treated with DOACs and VKAs (RR 0.70; 95% CI 0.34–1.44). Neither the risk of major bleeding (RR 0.84; 95% CI 0.32–2.19) nor the risk of non-major but clinically relevant bleeding (RR 0.92; 95% CI 0.62–1.36) differed between the treatment groups. Among the subgroups of patients with PC (*n* = 40 DOAC vs. 28 VKA), PS (*n* = 37 vs. 40), and AT (*n* = 15 vs. 11) deficiency only, the recurrence and bleeding event rates were low in both the DOAC and VKA groups. 

Hence, the combined results are encouraging and reassuring regarding DOACs in patients with thrombophilia, including those with severe thrombophilia. The American College of Chest Physicians 2021 guidelines [93] and the 2019 ESC guidelines [14] indicate that DOACs are recommended over VKAs to treat VTE but do not yet provide guidance for patients with thrombophilia due to limited data. However, the information on thrombophilia in these studies is not detailed regarding the type of deficiency (i.e., quantitative or qualitative) and, as described in Section 2, the thromboembolic risk also differs according to the type of deficiency and the genotypic characterization. Therefore, additional high-quality evidence in well-characterized thrombophilia patients is needed to clarify this point. 

## 5. Conclusions

Our knowledge on inherited thrombophilia has dramatically improved over the last few decades, leading to a better detection and characterization of thrombophilia and associated VTE risk. The rarest thrombophilia deficiencies are associated with a severe phenotype (AT, PC, and PS deficiencies) when compared to the more common genetic variants (FVL and F2 C.*97G>A). The widespread use of DOACs for VTD has raised new issues concerning inherited thrombophilia. Thrombophilia testing can be impacted by DOACs, which may cause false-negative results. Thrombophilia testing may be significantly affected by even small amounts of DOACs. Their effects are drug, concentration, test methodologic, and reagent dependent. AT activity can be studied with either FXa- or FIIa-based assays depending on the target of the DOAC. For PC and PS, apixaban apparently interferes less than rivaroxaban. PC clot-based assays would be less sensitive to DOACs, with possible testing at low concentrations that needs to be confirmed for each reagent. Therefore, current treatment information is mandatory to prevent misclassification and subsequent clinical consequences. Moreover, the adsorbent procedure appears to be an effective and simple way to overcome the interference of DOACs in coagulation tests and should soon facilitate the interpretation of thrombophilia screening tests in patients taking DOACs. In sum, inherited thrombophilia testing in DOAC-treated patients should be performed in an expert laboratory center for thrombophilia. Severe thrombophilia, associated with a higher risk of thrombosis recurrence, is rare and poorly represented in clinical studies. The available data reported in the literature are encouraging concerning DOAC efficacy and security in this setting. However, the thrombosis risk varies within deficiencies, which is why complementary evidence, including well-characterized thrombophilia patients, would be useful to confirm present data. 

## Figures and Tables

**Table 1:** Prevalence and associated risk of venous thromboembolism of inherited thrombophilia [15,16,17].

Inherited Thrombophilia	Prevalence in the General Population	Prevalence in Unselected Patients with VTE	RR for the First Episode of VTE *	RR for Recurrent VTE
*Severe thrombophilia*				
Antithrombin deficiency	0.02–0.2%	1.0%	≈15	1.9–2.6
FVL (homozygous)	0.02%	<2.0%	≈10	1.2 (0.5–2.6)
Double heterozygous (FVL and *F2* c.*97G>A)	<0.1%	<2.0%	≈10	(0.6–1.9)
Protein C deficiency	0.2–0.4%	3.0%	4.0–6.0	1.4–1.8
Protein S deficiency	0.03–0.5%	1.0–2.0%	1.0–10.0	1.0–1.4
*Mild thrombophilia*				
FVL (heterozygous)	3.0–7.0%	20.0%	3.0–5.0	1.2–1.4
*F2* c.*97G>A (heterozygous)	0.7–4.0%	6.0%	2.0–3.0	0.7–1.4

VTE: venous thromboembolism; RR: relative risk. * Notably, the first VTE may appear after 50 years in patients with severe thrombophilia.

**Table 2:** Plasma testing for the diagnosis of inherited thrombophilia.

Antithrombin Deficiency	Type I	Type IIHBS (Heparin-Binding Site)	Type IIRS (Reactive Site)	Type IIPE (Pleiotropic)
Heparin cofactor activity (FIIa- or FXa-based assay)	↓	↓	↓	↓/N
Progressive activity	↓	N	↓	↓/N
AT antigen	↓	N	N	↓/N
**Protein C Deficiency**	**Type I**	**Type 2a (IIAM)** **(Amidolytic)**	**Type 2b (IIAC)** **(Anticoagulant)**	
PC anticoagulant activity (clot-based assays)	↓	↓	↓	
PC amidolytic activity (chromogenic assays)	↓	↓	N	
PC antigen	↓	N	N	
**Protein S Deficiency**	**Type I**	**Type II** **(Qualitative)**	**Type III**	
PS activity (clot-based assays)	↓	↓	↓	
Free PS antigen	↓	N	↓	

AT: antithrombin; PC: protein C; PS: Protein S.

**Table 4:** Performance of DOAC neutralizing commercialized systems based on AC.

	DOAC-Stop^®^	DOAC Remove^®^	DOAC-Filter^®^
	***DOAC Neutralization***		
Samples tested	Patients treated with [57]: *n* = 30D (2–406 ng/mL) *n* = 26A (10–316 ng/mL) *n* = 10E (21–354 ng/mL) *n* = 27R (7–456 ng/mL) *n* = 3R, *n* = 1A [45]	Patients treated with [58]: *n* = 12A (80; 634 ng/mL) *n* = 9R (57; 922 ng/mL)	Normal pooled plasma spiked with DOAC about 300 ng/mL [66] Patients treated with [66]: *n* = 6D (30–439 ng/mL) *n* = 6A (147–298 ng/mL) *n* = 6R (34–295)
Results	Residual level < limit of quantification of the corresponding DOAC assays.	Residual level < limit of quantification of the corresponding DOAC assays except for two patients (1R 344 → 32 ng/mL) and 1A 510 → 95 ng/mL	Residual level < limit of quantification of the corresponding DOAC assays except 1A at 298 → 25.1 ng/mL
	***Impact on Natural Anticoagulant***		
Antithrombin	No impact [44,45,57,67]	No impact [56,67]	No impact [66]
Protein C	No impact [45,57,67]	No impact [56,58,67]	No impact [66]
Protein S	No impact [45,57,67]	No impact [56,58,67]	
	***Correction in DOAC-Treated Patients***		
Antithrombin	Anti-FXa activity (Innovance) in rivaroxaban- and apixaban-treated patients [44]		
Protein C		Protein C anticoagulant activity (Staclot Protein C) in rivaroxaban- and apixaban-treated patients [58]	
Protein S		Protein S anticoagulant activity (Staclot Protein S ) in rivaroxaban- and apixaban-treated patients [58]	

A, apixaban; D, dabigatran; E, edoxaban; R, rivaroxaban.

## Data Availability

Not applicable.
